# Expert panel consensus statement on the optimal use of pomalidomide in relapsed and refractory multiple myeloma

**DOI:** 10.1038/leu.2014.60

**Published:** 2014-03-04

**Authors:** M A Dimopoulos, X Leleu, A Palumbo, P Moreau, M Delforge, M Cavo, H Ludwig, G J Morgan, F E Davies, P Sonneveld, S A Schey, S Zweegman, M Hansson, K Weisel, M V Mateos, T Facon, J F S Miguel

**Affiliations:** 1 University of Athens School of Medicine, Athens, Greece; 2 Service des Maladies du Sang, Hôpital Huriez, CHRU Lille, Lille, France; 3 Divisione di Ematologia dell'Università di Torino, Azienda Ospedaliera S Giovanni Battista, Turin, Italy; 4 Service d'Hematologie, CHU, Nantes, France; 5 University Hospital Leuven, Leuven, Belgium; 6 Seràgnoli Institute of Hematology, Bologna University School of Medicine, Bologna, Italy; 7 1st Department of Internal Medicine, Center for Oncology and Hematology, Wilhelminenhospital, Vienna, Austria; 8 Institute of Cancer Research, Royal Marsden Hospital, London, UK; 9 Department of Hematology, University Hospital Rotterdam, Rotterdam, The Netherlands; 10 Department of Haemato-oncology, King's College Hospital and King's College London, London, UK; 11 Department of Hematology, VU University Medical Center, Amsterdam, The Netherlands; 12 Department of Hematology, Skåne University Hospital, Lund University, Lund, Sweden; 13 University of Tuebingen, Tuebingen, Germany; 14 Hospital Universitario de Salamanca, CIC, IBMCC (USAL-CSIC), Salamanca, Spain; 15 Clinica Universidad de Navarra, Centro Investigaciones Medicas Aplicada (CIMA), Pamplona, Spain

## Abstract

In this report, a panel of European myeloma experts discuss the role of pomalidomide in the treatment of relapsed and refractory multiple myeloma (RRMM). Based on the available evidence, the combination of pomalidomide and low-dose dexamethasone is a well-tolerated and effective treatment option for patients with RRMM who have exhausted treatment with lenalidomide and bortezomib. The optimal starting dose of pomalidomide is 4 mg given on days 1–21 of each 28-day cycle, whereas dexamethasone is administered at a dose of 40 mg weekly (reduced to 20 mg for patients aged >75 years). The treatment should continue until evidence of disease progression or unacceptable toxicity. Dose-modification schemes have been established for patients who develop neutropenia, thrombocytopaenia and other grade 3–4 adverse events during pomalidomide therapy. Guidance on the prevention and management of infections and venous thromboembolism is provided, based on the available clinical evidence and the experience of panel members. The use of pomalidomide in special populations, such as patients with advanced age, renal impairment or unfavourable cytogenetic features, is also discussed.

## Introduction

Despite recent treatment advances, multiple myeloma (MM) remains an incurable disease in the majority of patients. The management of patients who have already received multiple prior therapies poses a distinct clinical challenge.^1^ Because of the advanced nature of the disease, these patients often have significant disease-related comorbidity, such as thrombocytopaenia, bone disease or renal impairment,^2, 3^ as well as indications of marked immunosuppression.^2, 4, 5, 6, 7, 8^ Patients may have poor quality of life^9^ because of disease-related symptoms, adverse events from prior therapies or cumulative toxicity, such as impaired bone marrow reserve^2^ or neuropathy.^10^ Periods of remission become increasingly shorter with each subsequent therapy,^11^ and the prognosis for patients who have exhausted treatment with immunomodulatory drugs (thalidomide or lenalidomide) and bortezomib is poor: the expected median event-free survival is 5 months and median overall survival (OS) is 9 months.^1^ Thus, there is an unmet need for effective and well-tolerated novel antimyeloma therapies that improve outcomes in patients with advanced myeloma.

Pomalidomide (Imnovid, Celgene Europe Ltd, Uxbridge, UK; Pomalyst, Celgene Corporation, Summit, NJ, USA) is an IMiDs(R) immunomodulatory compound that has demonstrated activity in MM patients with disease refractory to lenalidomide and bortezomib.^12, 13, 14, 15, 16^ Pomalidomide was approved by the FDA (Food and Drug Administration) in February 2013 and the EMA (European Medicines Agency) in August 2013 for use alone (in the United States) or in combination with dexamethasone in patients with MM who have received at least two prior therapies including lenalidomide and bortezomib and have demonstrated disease progression on their last therapy^17^ (within 60 days of the last treatment for the United States).^18^ The aim of this review is to provide practical guidance to help haematologists and oncologists maximise efficacy and minimise safety risks through appropriate dosing, monitoring and intervention for adverse events with pomalidomide treatment.

## Pomalidomide

### Mechanism of action

Pomalidomide is a distinct IMiDs(R) immunomodulatory compound with multiple cellular effects that inhibit the growth of myeloma cells.^19^ Pomalidomide has direct effects on myeloma cells by inhibiting their growth and survival,^20, 21, 22, 23, 24, 25, 26, 27, 28, 29, 30^ and it also inhibits stromal support from the bone marrow microenvironment that can promote myeloma cell growth.^2, 31, 32, 33, 34, 35, 36, 37, 38, 39^ In addition, pomalidomide has potent immunomodulatory effects that enhance the immune response to myeloma cells by stimulating natural killer cells^40, 41, 42^ and by inhibiting regulatory T cells.^43^ Recent evidence suggests that the effects of pomalidomide may be partially mediated by cereblon, a component of the E3 ubiquitin ligase complex.^44, 45, 46^ Preclinical data indicate that pomalidomide is active in drug-resistant myeloma cell lines,^44, 47^ including lenalidomide-resistant cells,^48, 49^ and produces synergistic effects when combined with dexamethasone.^50^

### Efficacy in clinical trials

In a phase I/II study (MM-002), the combination of pomalidomide and low-dose dexamethasone was assessed in patients with relapsed and refractory multiple myeloma (RRMM) who had received prior lenalidomide and bortezomib.^14, 51^ The median number of prior therapies was 5 (range 1–13); all patients had received prior steroids, lenalidomide and bortezomib, and 62% were refractory to both lenalidomide and bortezomib.^14^ In phase I of the study, the dose-limiting toxicity (grade 4 neutropenia) occurred at a pomalidomide dose of 5 mg; the maximum tolerated dose of pomalidomide was, therefore, 4 mg given on days 1–21 of each 28-day cycle in combination with low-dose dexamethasone (40 mg weekly for patients ⩽75 years; 20 mg weekly for patients >75 years).^51^ With this dose and schedule and in the pivotal phase II trial, pomalidomide plus low-dose dexamethasone was associated with an overall response rate of 33% and median duration of response of 8.3 months. With a median follow-up of 14.2 months, the median progression-free survival (PFS) and OS were 4.2 and 16.5 months, respectively.^14^ In comparison, response to single-agent pomalidomide was 18% with a median duration of response, PFS and OS of 10.7, 2.7 and 13.6 months, respectively, indicating that the addition of low-dose dexamethasone to pomalidomide improves efficacy.^14^ Subanalyses indicated that the efficacy of pomalidomide plus low-dose dexamethasone was similar regardless of whether the patient was refractory to lenalidomide or bortezomib as last prior therapy.^52^

In a randomised phase II trial (IFM 2009-02), two different schedules of pomalidomide administration were compared in combination with low-dose dexamethasone (40 mg weekly) in patients who were refractory to, or had never achieved a response to, lenalidomide and bortezomib.^15^ Patients received pomalidomide (4 mg) for either 21 days or 28 days of each 28-day cycle, plus dexamethasone, using a similar schedule as in the MM-002 trial. The schedule of administration did not affect the overall response rate (35% vs 34%) or time to progression (TTP; 5.4 months overall), and the median PFS and OS for the total study population were 4.6 and 14.9 months, respectively. The investigators concluded that the 21/28-day schedule is preferred because it provided equivalent efficacy while facilitating long-term management by requiring less growth- factor support compared with the 28/28-day schedule (median number of treatment cycles: 8 vs 6). Notably, the TTP achieved with pomalidomide plus low-dose dexamethasone in responding patients was better than that achieved with the last prior therapy before entering the study,^15^ suggesting that pomalidomide may have the potential to change the natural course of the disease (characterised by progressively shorter remissions with each subsequent treatment).^11^

In a randomised and pivotal phase III trial (MM-003), pomalidomide plus low-dose dexamethasone was compared with high-dose dexamethasone in patients who had failed both bortezomib and lenalidomide treatment.^16^ With a median follow-up of 10 months, pomalidomide plus low-dose dexamethasone decreased the rate of progression by 52% (PFS 4.0 vs 1.9 months; hazard ratio (HR)=0.48; 95% confidence interval (CI) 0.39–0.60; *P*<0.001) and significantly improved OS (12.7 vs 8.1 months; HR=0.74; 95% CI 0.56–0.97; *P*=0.03). However, the impact on OS is probably underestimated because of the confounding effect of crossover that occurred in the high-dose dexamethasone arm. At the time of the analysis, 50% of patients assigned to high-dose dexamethasone had received pomalidomide, and by month 16 of study treatment, it was estimated that all patients assigned to high-dose dexamethasone would have received pomalidomide as salvage therapy, underscoring the superiority of the combination regimen.^16^ Similar efficacy was seen for pomalidomide plus low-dose dexamethasone vs high-dose dexamethasone in patients with disease refractory to both lenalidomide and bortezomib (PFS 3.7 vs 2.0 months; HR=0.52; *P*<0.001 and OS 11.1 vs 7.7 months; HR=0.77; *P*=0.096) and in those who received lenalidomide as last prior therapy (PFS 4.6 vs 1.9 months; HR=0.38; *P*<0.001 and OS 12.3 vs 7.3 months; HR=0.53; *P*=0.01).^16^

Data from these large multicentre trials support the use of pomalidomide plus low-dose dexamethasone in patients with RRMM who have received prior lenalidomide and bortezomib. Practical issues regarding the optimal use of pomalidomide in this setting are discussed below.

## Candidates for pomalidomide plus low-dose dexamethasone therapy

**Figure 1:**
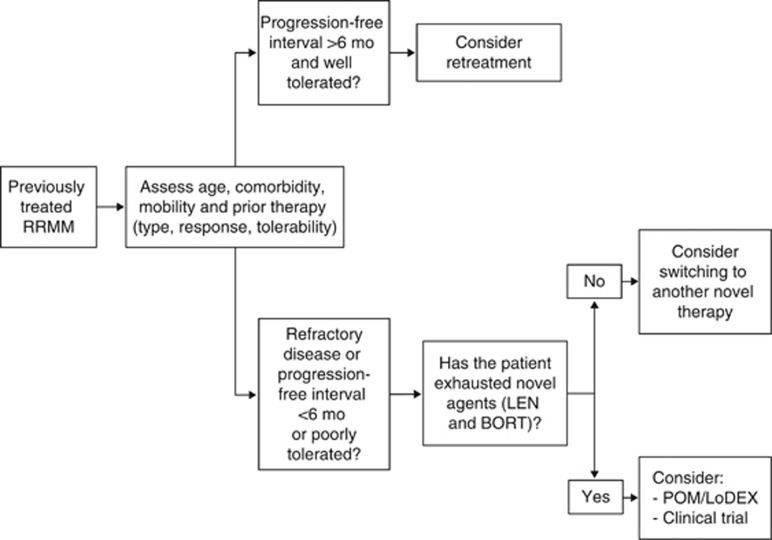
Treatment algorithm for patients who have failed prior therapy for RRMM. BORT, bortezomib; LEN, lenalidomide; POM/LoDEX, pomalidomide plus low-dose dexamethasone.

Determining whether a patient is eligible for treatment with pomalidomide plus low-dose dexamethasone requires careful consideration of multiple factors, particularly the type of prior therapy, the quality of response and the tolerability of the prior therapy (Figure 1).^53^ In general, for patients who respond to prior therapy, tolerate it well and have a period of unmaintained remission not inferior to the median TTP or PFS expected with prior therapy, retreatment may be considered. For patients who have a poor response to prior therapy or do not tolerate it well, switching to an alternative novel agent is recommended.^53^ For patients who have exhausted novel therapies (lenalidomide and bortezomib), treatment options include pomalidomide plus low-dose dexamethasone or enrolment in a clinical trial or palliative care.^53^ The proteasome inhibitor carfilzomib is approved in the United States but not currently approved for use in the European Union.

Candidates for pomalidomide plus low-dose dexamethasone therapy must have received at least two prior therapies and have documented disease progression on their last therapy.^17^ In the MM-003 trial, the proportion of patients treated with pomalidomide plus low-dose dexamethasone who had disease refractory to lenalidomide, bortezomib or both was 95%, 79% and 75%, respectively,^16^ per available hospital records of prior treatment. The trial also stratified the patients based on patient-reported data at study entry. Most patients (82%) were classified as having disease refractory to lenalidomide and bortezomib, whereas smaller proportions of patients were intolerant to bortezomib (15%) or had relapsed on lenalidomide and/or bortezomib and were refractory to any subsequent therapy (3%). In the clinical setting, appropriate candidates for pomalidomide therapy are, therefore: patients who have received prior lenalidomide and bortezomib and have become refractory to these agents; patients who have relapsed on lenalidomide and/or bortezomib and are refractory to their subsequent therapy; and patients unresponsive or intolerant to existing agents.

## Optimal dose and schedule

### Optimal pomalidomide dose and schedule

Based on the available clinical evidence, the recommended starting dose of pomalidomide is 4 mg given on days 1–21 of each 28-day cycle until disease progression.^17^ This dose was used in the MM-002 trial,^14, 51^ MM-003 trial^16^ and IFM 2009-02 trial^15^ and represents the most robust data on pomalidomide currently available in RRMM. Early studies explored various doses and schedules of pomalidomide.^54, 55^ This led to a series of cohort studies conducted by the Mayo Clinic (Rochester, MN, USA) using pomalidomide doses of 2 or 4 mg given either continuously or for 21 days of each 28-day cycle;^12, 13, 56, 57^ however, these were not dose-finding studies. In the phase I dose-escalation portion of the MM-002 trial, response and duration of response tended to increase with increasing doses of pomalidomide, and the maximum tolerated dose was determined to be 4 mg when given for 21 days of each 28-day cycle.^51^ In the IFM 2009-02 trial, pomalidomide 4 mg with a less intensive 21/28-day schedule, rather than continuous dosing, was recommended for further investigation, because the 1-week treatment break allowed for bone marrow recovery, leading to improved tolerability.^15^

Based on clinical trial experience, RRMM patients should have adequate blood cell counts, including absolute neutrophil count ⩾1000/μl before starting pomalidomide plus low-dose dexamethasone.^16^ Platelet count should be ⩾75 000/μl if <50% of bone marrow nucleated cells are plasma cells or ⩾30 000/μl if ⩾50% of bone marrow nucleated cells are plasma cells.^16^ For patients with absolute neutrophil count and blood cell counts below these thresholds, pomalidomide plus low-dose dexamethasone may be considered with the provision of adequate growth factor support and platelet transfusion.


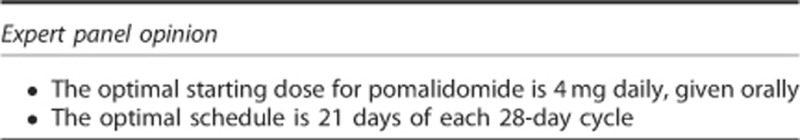


### Optimal dexamethasone dose and schedule

High-dose dexamethasone (40 mg daily on days 1–4, 9–12 and 17–20 of each 28-day cycle) is often used as rescue therapy in heavily pretreated patients with RRMM, and has been used as a comparator in registrational studies of novel therapies, including lenalidomide^58, 59^ and bortezomib.^60^ However, evidence suggests that low-dose dexamethasone (40 mg weekly) may be more effective and better tolerated than high-dose dexamethasone when combined with lenalidomide in newly diagnosed patients.^61^ The three main trials of pomalidomide in RRMM (MM-002, MM-003 and IFM 2009-02) therefore evaluated low-dose dexamethasone in combination with pomalidomide. Notably, in all three trials, the dose of dexamethasone was reduced to 20 mg weekly in patients aged >75 years to improve the tolerability of the regimen for these patients, particularly with regard to the risk of infection. In clinical practice, patients' vulnerabilities, comorbidities and age, both biological and chronological, should also be examined before initiating dexamethasone.^62^ Some experts recommend a lower age threshold of 70 years.


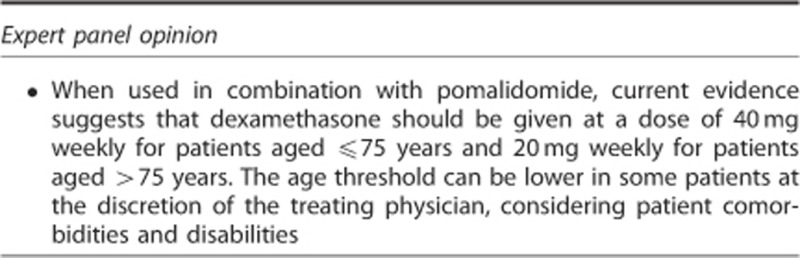


### Optimal duration of therapy

In clinical trials, pomalidomide plus low-dose dexamethasone therapy was given until evidence of disease progression or unacceptable toxicity, and it is recommended to continue pomalidomide plus low-dose dexamethasone treatment until disease progression.^17^ In the IFM 2009-02 trial, 10 of 85 patients (12%) received therapy for more than 30 months, supporting the long-term safety of pomalidomide-based therapy.^63^ Currently, there is no evidence to support stopping or reducing the dose of pomalidomide in responding patients. However, it is the opinion of the panel members that reducing the dose of dexamethasone may be considered to improve long-term tolerability, based on the known safety profile of dexamethasone and experience with lenalidomide and dexamethasone.^61^ An ongoing Australian randomised trial evaluating four courses of pomalidomide plus dexamethasone followed by either pomalidomide monotherapy or pomalidomide plus dexamethasone will provide useful information on the duration of dexamethasone therapy in nonprogressing patients.^64^


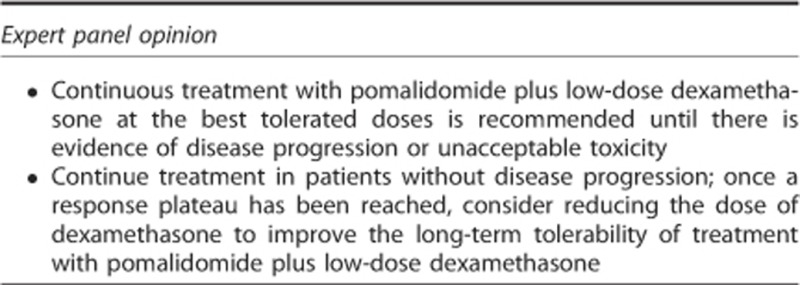


## Managing adverse events

The types of adverse events seen with pomalidomide are similar to those seen with lenalidomide. The most common grade 3–4 adverse event associated with pomalidomide plus low-dose dexamethasone is myelosuppression. In clinical trials, the incidence of grade 3–4 neutropenia, thrombocytopaenia and anaemia ranged from 41 to 62%, from 19 to 27% and from 22 to 36%, respectively.^15, 16, 65^ Neutropenia and other adverse events of clinical interest, as well as recommended dose adjustments for pomalidomide, are discussed below.

### Neutropenia

Based on clinical experience with lenalidomide,^58, 59^ neutropenia was anticipated to be a primary adverse event of pomalidomide therapy. Indeed, neutropenia is one of the most common adverse events observed in patients treated with pomalidomide plus low-dose dexamethasone.^15, 16, 65^ The reported incidence of grade 3–4 neutropenia with pomalidomide plus low-dose dexamethasone ranges from 41 to 62%.^15, 16, 65^ In MM-003, 26% of patients developed grade 3 neutropenia and 22% had grade 4 neutropenia,^16^ with most cases occurring within the first few cycles of therapy.^66^ Few patients (<10%) experienced febrile neutropenia.^16, 65^

Depending on its severity, neutropenia may be managed with dose interruptions, dose modifications and/or growth factor support. In MM-002, 46% of all patients treated with pomalidomide plus low-dose dexamethasone received growth factor support; most cases of neutropenia resolved (49 of 55 patients with any grade neutropenia (89%)) in the pomalidomide plus low-dose dexamethasone group after a median of 1.4 months.^65^ In MM-003, the median time to onset of neutropenia was 0.7 months (range 0.03–8.7 months).^66^ Growth factor support was used in 43% of patients treated with pomalidomide plus low-dose dexamethasone,^16^ whereas 23% of patients required pomalidomide dose interruption because of neutropenia, and only 8% required a reduction in pomalidomide dose.^66^

**Figure 2:**
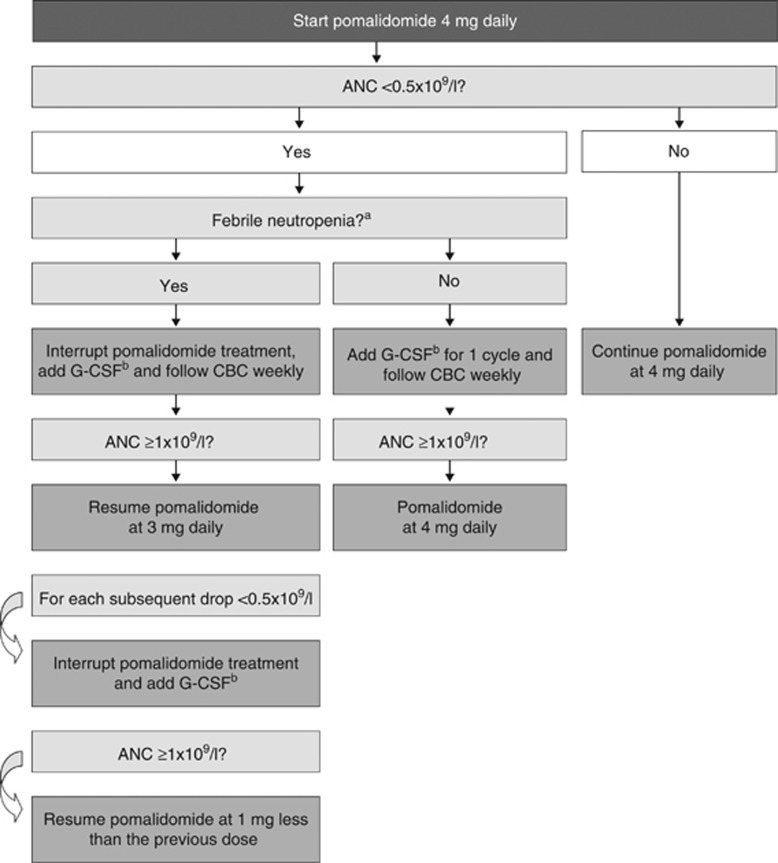
Recommended pomalidomide dose modifications for neutropenia.^17^ The minimum blood levels required to start treatment with pomalidomide at the full dose of dose 4 mg are ANC ⩾1 × 10^9^/l; platelets ⩾75/10^9^/l or ⩾30 if ⩾50% of bone marrow nucleated cells are plasma cells. It is not recommended to give pomalidomide in doses <1 mg. ANC, absolute neutrophil count; CBC, complete blood count; G-CSF, granulocyte colony-stimulating factor. ^a^Febrile neutropenia is defined as fever ⩾38.5 °C and ANC <1 × 10^9^/l. ^b^G-CSF cycle; 300 μg/kg for 3 days (days 22–24 of each 28-day cycle).

**Figure 3:**
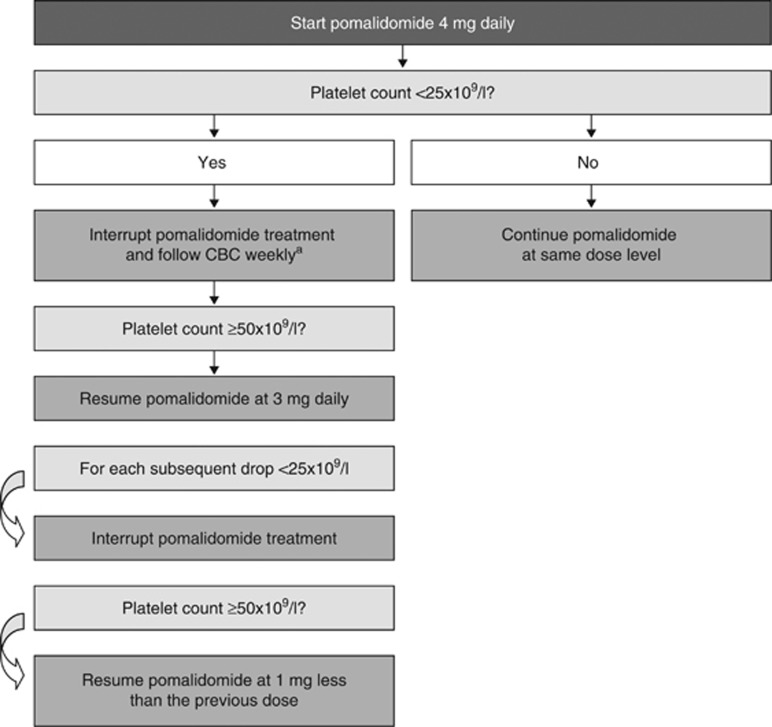
Recommended pomalidomide dose modifications for thrombocytopaenia.^17^ The minimum blood levels required to start treatment with pomalidomide at the full dose of dose 4 mg are ANC ⩾1 × 10^9^/l; platelets ⩾75/10^9^/l or ⩾30 if ⩾50% of bone marrow nucleated cells are plasma cells. It is not recommended to give pomalidomide in doses <1 mg. CBC, complete blood count. ^a^Consider frequent platelet transfusions.

Given the inherent risk of infection in patients with RRMM,^4, 67^ those treated with pomalidomide should be monitored closely for neutropenia and other haematological adverse events. Unlike the long-lasting neutropenia associated with cytotoxic chemotherapy, the neutropenia with pomalidomide is usually short-lived. Recommended dose modifications for pomalidomide-related neutropenia and thrombocytopaenia have been established (Figures 2 and 3, respectively). Growth factors may be considered for patients who develop neutropenia during pomalidomide therapy.^17^ There is limited evidence available regarding prophylactic use of growth factors in patients receiving pomalidomide. For lenalidomide, some evidence suggests that prophylactic use of granulocyte colony-stimulating factor (300 μg/kg for 3 days (days 22–24 of each 28-day cycle)) during the first few treatment cycles may reduce the risk of further neutropenia, treatment delays, dose modifications and infection.^68, 69^ Guidelines have been developed on the use of growth factor support in cancer patients in general^70, 71, 72, 73^ and for RRMM patients receiving lenalidomide therapy.^69^ Based on the above data, prophylactic granulocyte colony-stimulating factor administration (that is, twice a week) could be considered in patients with baseline extensive bone marrow involvement and/or low neutrophil count.


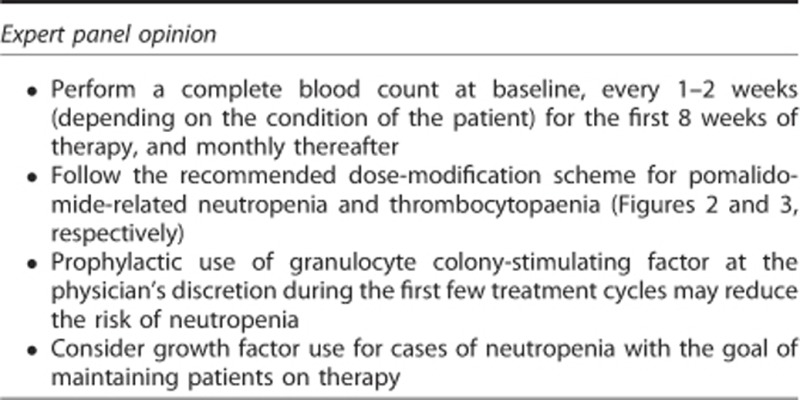


### Infection

Increased susceptibility to bacterial infection is one of the most common clinical problems in patients with myeloma: >75% of patients will develop a serious infection at some time in the course of the disease.^74^ The most common types of infection are respiratory and urinary tract infections; the most frequently encountered pathogens are *Streptococcus pneumoniae*, *Haemophilus influenza*, *Staphylococcus aureus* and *Klebsiella pneumoniae* in the lungs and *Escherichia coli* and other Gram-negative bacteria in the urinary tract.^74^ Various factors contribute to the increased risk of infection. Disease-related factors include B-cell dysfunction (manifested as hypogammaglobulinaemia), low CD4+ cell count and natural killer cell dysfunction, all of which contribute to immunodeficiency that is often present even in untreated myeloma patients.^4, 67^ The advanced age of most myeloma patients (the median age at diagnosis is ∼70 years^75^) is associated with reduced physiological reserve of various organ systems and several physical, cognitive and social conditions that can predispose patients to opportunistic infections.^67^ Lastly, patients with RRMM may develop cumulative immunodeficiency after exposure to multiple therapies that increase infection risk.^67^

Infection is a commonly reported adverse event in trials of pomalidomide. In IFM 2009-02, 23% of patients had grade 3–4 infection (19% with the 21/28-day schedule and 27% with 28/28-day schedule).^15^ In MM-003, grade 3–4 infection occurred in 30% and 24% of patients treated with pomalidomide plus low-dose dexamethasone and high-dose dexamethasone, respectively.^16^ The reported incidence of grade 3–4 pneumonia ranged from 13 to 22%.^15, 16, 51, 65^ Notably, the occurrence of neutropenia in MM-003 did not appear to affect the incidence of infection, and most infections occurred in the absence of neutropenia (66% with pomalidomide plus low-dose dexamethasone vs 86% with high-dose dexamethasone); few patients (2%) discontinued treatment because of infection.^16^

Given the increased risk of infection, routine vaccinations are recommended for the patients and their contacts, and antibiotic prophylaxis should be considered for all patients receiving pomalidomide.^53, 71^ Prophylaxis should be given for a minimum of the first 3 months of therapy, when the risk of infection is the highest.^53, 69^ For patients at high risk of infection, such as those with low blood counts or a previous history of infection or both, antibiotic prophylaxis is recommended for the complete duration of pomalidomide plus dexamethasone therapy. An optimal antibiotic prophylaxis regimen has not been determined, but several options are available (trimethoprim–sulfamethoxazole, quinolones, penicillin, amoxicillin and so on); clinicians should follow standard protocols as established by their institution.^53, 71^ Caution is warranted when using the quinolones ciprofloxacin and enoxacin because they strongly inhibit the activity of CYP1A2, the cytochrome *P*450 isoform primarily responsible for the metabolism of pomalidomide (Table 1) and can therefore increase exposure to pomalidomide.^76^ If these quinolones are administered concurrently with pomalidomide, patients should be monitored closely for adverse events. Importantly, the quinolones norfloxacin, ofloxacin, levofloxacin, moxifloxacin and gemifloxacin have little or no effect on CYP1A2 (refs. 77,78) and are therefore not expected to affect pomalidomide metabolism; these agents are preferred when quinolones must be administered concurrently with pomalidomide.

For patients who develop infection during pomalidomide therapy, treatment should be interrupted (regardless of the presence or absence of neutropenia) until the infection resolves and then may be restarted. Treatment of infection in patients receiving pomalidomide should follow standard protocols and include immediate empirical antibiotic treatment that may be adapted as needed, when results from blood cultures and other tests become available.^53, 71^


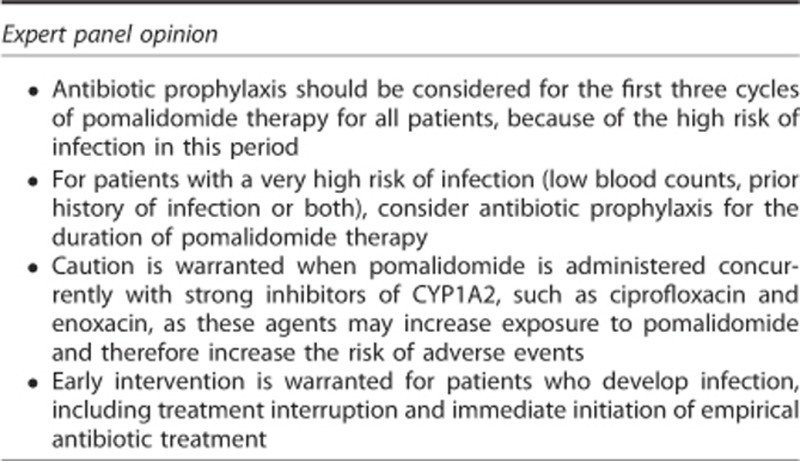


### Venous thromboembolism (VTE)

VTE, including deep vein thrombosis and pulmonary embolism, is a rare but potentially serious adverse event that has been reported with immunomodulatory drug therapy.^58, 59, 79, 80^ In both MM-002 and MM-003, the reported incidence of deep vein thrombosis in patients treated with pomalidomide plus low-dose dexamethasone was 2%, although thromboprophylaxis was given,^14, 16^ and, in IFM 2009-02, the incidence was 4% among those who received aspirin prophylaxis.^15^ The incidence of deep vein thrombosis in patients treated with pomalidomide appears to be lower than that reported historically for lenalidomide,^58, 59^ although this may reflect increased thromboprophylaxis use that was mandatory in trials of pomalidomide.

**Figure 4:**
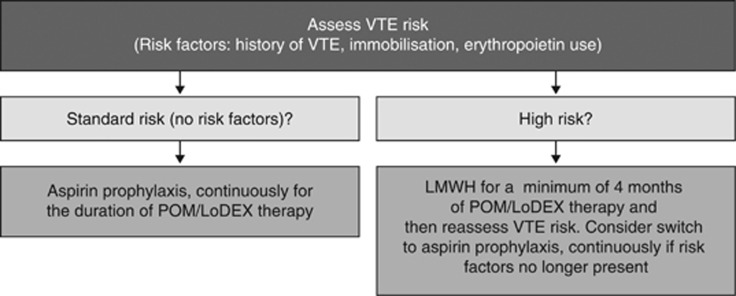
Recommendations for determining appropriate thrombosis prophylaxis in patients with RRMM treated with POM/LoDEX. LMWH, low-molecular-weight heparin; POM/LoDEX, pomalidomide plus low-dose dexamethasone; VTE, venous thromboembolism.

All patients with MM treated with immunomodulatory drugs, including pomalidomide, should receive thromboprophylaxis. The optimal approach to thromboprophylaxis has not been established, but it is generally agreed that the type of thromboprophylaxis selected depends on the risk of VTE, as determined by the individual patient's characteristics and the proposed treatment regimen.^79, 80, 81^ Aspirin prophylaxis is generally recommended for patients with standard risk of VTE, and low-molecular-weight heparin (prophylactic dose) or vitamin K antagonists (international normalised ratio 2–3) are recommended for patients with high risk of VTE (Figure 4). Several risk factors for VTE have been identified for patients with cancer^82^ and for MM specifically.^81^ Possible risk factors include treatment-related factors (high-dose dexamethasone, doxorubicin, multi-agent chemotherapy, IMiDs immunomodulatory compounds, erythropoietin) and patient-related factors (obesity, prior VTE, central venous catheter or pacemaker, infection, immobilisation, surgery, trauma, organ dysfunction, blood-clotting disorders).^82, 83^ In a large multicentre observational study (MELISSE), factors that predicted VTE in MM patients treated with immunomodulatory drugs (thalidomide or lenalidomide) include shorter time from diagnosis and concomitant use of erythropoietin.^79^ Evidence also suggested that development of deep vein thrombosis was unlikely to negatively affect survival outcomes.^79^ In 200 consecutive MM patients treated with lenalidomide at a single institution, the incidence of VTE was higher in previously untreated patients than in RRMM patients (9.4% vs 4.5%); among RRMM patients, the incidence of VTE was increased in patients aged >65 years compared with younger patients (8.1% vs 1.6%), despite increased use of low-molecular-weight heparin or vitamin K antagonists in older patients.^84^ In the 108 patients who received aspirin prophylaxis, a single-nucleotide polymorphism in the *NFkB1* gene was identified that was associated with increased risk of VTE.^84^ From a practical point of view, in patients treated with pomalidomide and low-dose dexamethasone, the main high-risk features for VTE are: a history of prior VTE, immobilisation and concomitant use of an erythropoiesis-stimulating agent. For patients who develop VTE during pomalidomide-based therapy, treatment should be interrupted temporarily and anticoagulation therapy should be initiated.^65^ Treatment may be resumed, probably within a few weeks, although the optimal timing has not been determined.


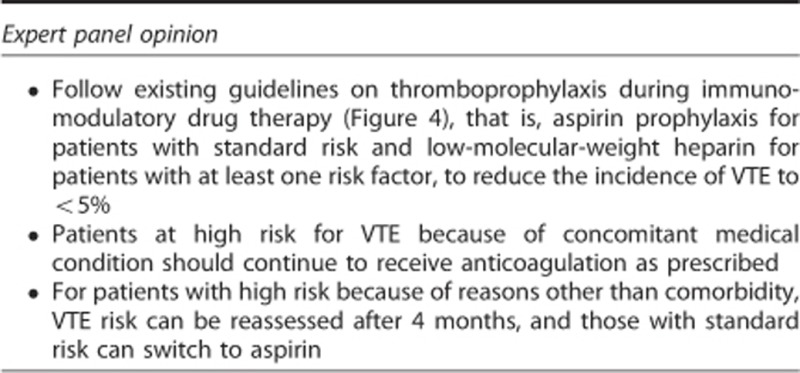


### Peripheral neuropathy

Peripheral neuropathy is a common and potentially treatment-limiting adverse event associated with thalidomide and bortezomib, but lenalidomide does not appear to cause substantial neurotoxicity.^85^ Although patients with grade ⩾2 peripheral neuropathy were excluded from pomalidomide trials, the incidence of newly occurring or worsening peripheral neuropathy during pomalidomide treatment was low. In MM-002, no cases of grade 3–4 peripheral neuropathy were observed despite the fact that 73% of patients in the pomalidomide plus low-dose dexamethasone arm had a history of peripheral neuropathy.^65^ In MM-003, 15% of patients treated with pomalidomide plus low-dose dexamethasone developed any-grade treatment-emergent peripheral neuropathy; 52% of these cases had grade 1 peripheral neuropathy at baseline.^66^ The incidence of grade 3–4 peripheral neuropathy was ⩽2% in both treatment arms.^16^

**Figure 5:**
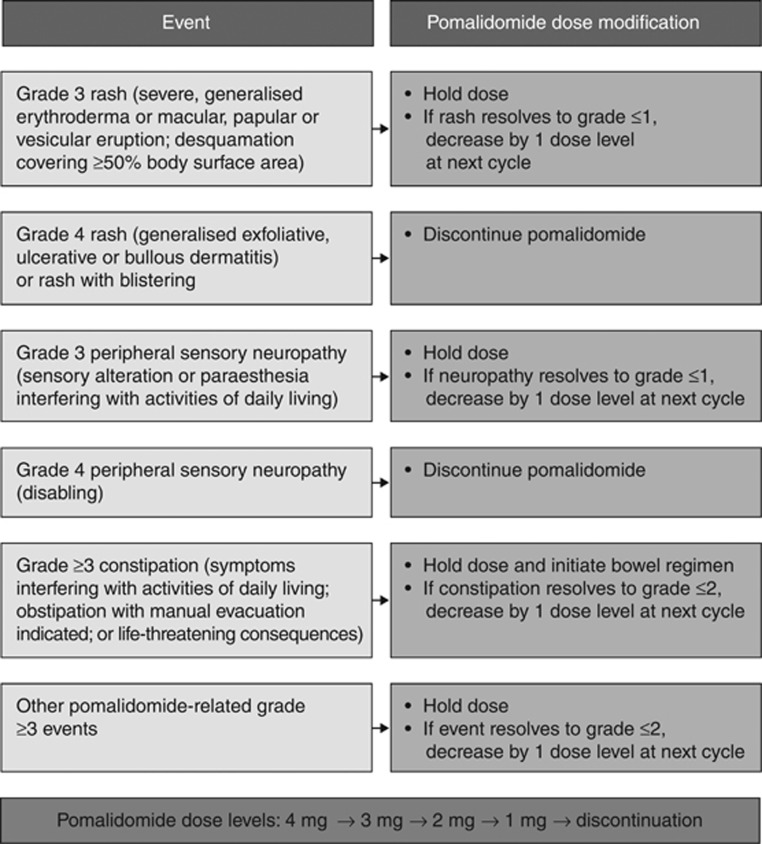
Recommended pomalidomide dose modifications for other nonhaematological adverse events.^65, 66^

For patients who develop grade 3 peripheral neuropathy, pomalidomide treatment should be interrupted, but may be resumed at a lower dose if the neuropathy resolves to grade 0–1; pomalidomide should be discontinued in patients who develop grade 4 peripheral neuropathy (Figure 5).^65, 66^


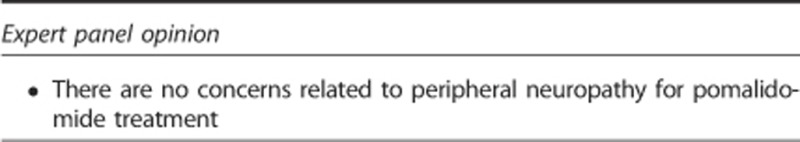


### Other adverse events

Other clinically relevant nonhaematological adverse events observed with pomalidomide plus low-dose dexamethasone include fatigue, gastrointestinal disorders, muscle cramps and rash. In MM-003, fatigue was reported in 34% of patients, although only 5% had grade 3 fatigue and no cases of grade 4 fatigue were observed.^16^ Any-grade diarrhoea, constipation and nausea occurred in 22%, 22% and 15% of patients, respectively, but grade 3 events were uncommon (1%, 2% and 1%, respectively). Muscle cramps and rash, which occurred in 16% and <10% of patients treated with pomalidomide plus low-dose dexamethasone, respectively, appear to be less common with pomalidomide than with lenalidomide.^58, 59^ Acute pulmonary toxicity is a rare, but serious, adverse event that manifests itself as acute interstitial pneumonitis. This complication needs to be recognised and treated promptly with corticosteroids.^86^

In most cases, nonhaematological grade 1–2 adverse events can be managed with standard interventions; patients with grade 3–4 adverse events may require pomalidomide dose modifications or discontinuation, as described in Figure 5. Anecdotally, calcium and magnesium supplementation or quinine may ameliorate muscle cramps. Caution is warranted when using pomalidomide in patients who developed rash during prior treatment with thalidomide or lenalidomide. For patients with mild-to-moderate maculopapular eruption or erythema, treatment with low-dose prednisone and antihistamines may be considered; for those with symptomatic and generalised rash, pomalidomide dose delay or reduction should be considered. Pomalidomide should be discontinued in the rare but severe cases of generalised exfoliative, ulcerative or bullous dermatitis.


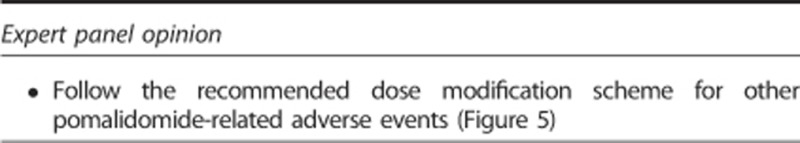


## Impact on quality of life

In MM-003, quality-of-life scores for Global Health Status and Physical Functioning worsened significantly by cycle 2 in patients treated with high-dose dexamethasone.^87^ In comparison, treatment with pomalidomide plus low-dose dexamethasone significantly prolonged the time to worsening of Global Health Status and Physical Functioning scores. In addition, fewer patients treated with pomalidomide plus low-dose dexamethasone experienced worsening in Fatigue scores, compared with those treated with high-dose dexamethasone. These findings suggest that, in terms of overall quality of life, the improved antimyeloma efficacy of pomalidomide plus low-dose dexamethasone may outweigh the negative impact of treatment-related adverse events compared with high-dose dexamethasone.

## Use in special populations

### Patients with renal impairment

Lenalidomide is mainly eliminated unchanged via the kidneys, and renal impairment has been shown to reduce clearance of lenalidomide. As a result, lenalidomide dose adjustments are needed in patients with renal impairment to avoid excessive and potentially toxic levels of the drug.^3, 88^ In contrast, pomalidomide is metabolised extensively before excretion; only 2% of pomalidomide is excreted unchanged in the urine, compared with 82% of lenalidomide.^76^ Compared with lenalidomide, pomalidomide also has a slower rate of absorption and a longer elimination phase in healthy volunteers and in patients with MM (Table 1). It is therefore hypothesised that renal function will not have a major effect on drug exposure.^89^

The available clinical data support the efficacy and safety of pomalidomide plus low-dose dexamethasone in patients with creatinine clearance ⩾45 ml/min using the standard starting doses. In a subanalysis of data from MM-002 in which patients were classified according to creatinine clearance, the safety and efficacy of pomalidomide plus low-dose dexamethasone appeared similar irrespective of the degree of renal function.^52, 90^ A preplanned renal assessment in MM-003 showed that treatment with pomalidomide plus low-dose dexamethasone significantly extended PFS and OS compared with high-dose dexamethasone in patients with or without moderate renal impairment.^91, 92^ However, these results should be interpreted with caution because the number of patients in each subgroup was small, and patients with severe renal impairment (serum creatinine >3 mg/dl in MM-002 and creatinine clearance <45 ml/min in MM-003) were excluded from these studies.^16, 52, 90^ An ongoing phase I study (MM-008) will help determine the optimal dose of pomalidomide when given with low-dose dexamethasone in patients with severe renal impairment (creatinine clearance <30 ml/min).^89, 93^ Preliminary data from this study indicate that pomalidomide plus low-dose dexamethasone is well tolerated, and dose escalation continues. A larger, phase IV study (MM-013) has been initiated in Europe to assess the safety and efficacy of pomalidomide plus low-dose dexamethasone in patients with RRMM and moderate-to-severe renal impairment, including those undergoing dialysis.


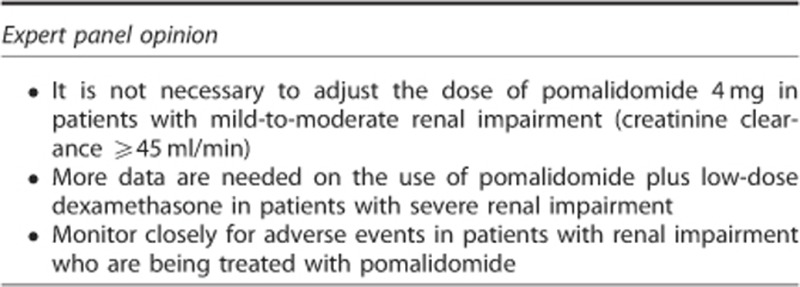


### Patients with unfavourable cytogenetics

In MM-002, pomalidomide plus low-dose dexamethasone showed promising activity in the subset of patients with unfavourable cytogenetics (del[17p] and/or t[4;14])^94^ and, in MM-003, treatment with pomalidomide plus low-dose dexamethasone resulted in significantly better PFS and OS than with high-dose dexamethasone, regardless of cytogenetic risk group.^95^ However, in IFM 2009-02, the presence of high-risk cytogenetics (del[17p] and/or t[4;14]) negatively affected outcomes: the 1-year OS rate in this cohort was 27% compared with 67% in patients with no cytogenetic abnormalities.^15^ In a phase II confirmatory trial (IFM 2010-02) evaluating pomalidomide plus low-dose dexamethasone in 50 patients with del(17p) and/or t(4;14), the median TTP and OS were 3 and 12 months, respectively.^96^ Notably, patients with del(17p) appeared to benefit more from treatment with pomalidomide plus low-dose dexamethasone than did those with t(4;14): the median TTP was 8 months vs 3 months and median OS was not reached vs 9 months, respectively. In another phase II study of 71 patients with high-risk disease, defined by gene-expression profiling (GEP70 or GEP80 signatures), increased lactate dehydrogenase levels or metaphase cytogenetic abnormalities, pomalidomide-based therapy had good antimyeloma activity: 28% of patients had a partial response or better; 82% had stable disease or better; and the 1-year PFS and OS rates were 13% and 63%, respectively.^97^ These data need to be interpreted with caution because of the relatively small number of patients, and additional data are needed.


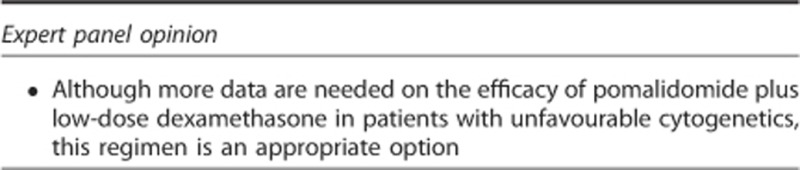


### Patients with advanced age

In IFM 2009-02, response to pomalidomide plus low-dose dexamethasone was not affected by age, and its safety profile in the subset of patients aged ⩾65 years (*n*=26) was acceptable.^15^ Survival outcomes were generally less favourable in older patients in both arms, compared with younger patients, as is usually the case in myeloma patients. Similarly, in MM-002, response to pomalidomide plus low-dose dexamethasone was not affected by age, and the safety profile was generally similar in patients aged ⩽65 and >65 years.^98, 99^ The incidence of pneumonia, however, was increased in older patients (29% vs 16%), underscoring the need for appropriate antibiotic prophylaxis in patients at risk of pneumonia. In MM-003, a subanalysis indicated that the efficacy and safety profiles of pomalidomide plus low-dose dexamethasone were not affected by age (<65 vs ⩾65 years).^16^ It should be noted that in MM-002 and MM-003, a reduced dose of dexamethasone (20 mg weekly) was used in patients >75 years of age^14, 16^ as is generally recommended for older patients with MM.^75^ Future trials should assess the use of alternative steroids (that is, prednisone) in elderly myeloma patients treated with pomalidomide.


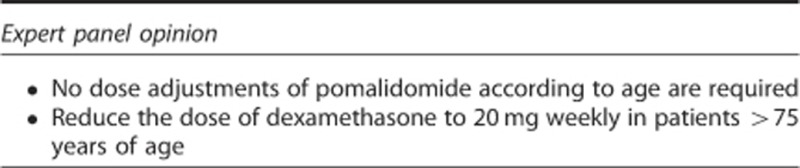


## Conclusions and future directions

**Figure 6:**
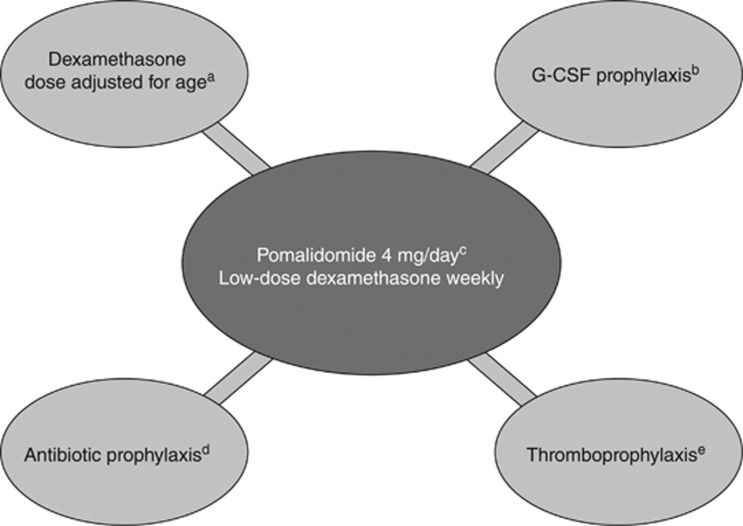
Considerations for initiation of pomalidomide plus low-dose dexamethasone therapy. G-CSF, granulocyte colony-stimulating factor; VTE, venous thromboembolism. ^a^Patients >75 years of age should receive dexamethasone 20 mg weekly; younger patients should receive a dose of 40 mg weekly. ^b^G-CSF use may be considered for the first three cycles to prevent neutropenia. ^c^The recommended starting dose of pomalidomide is 4 mg/day, regardless of the presence of comorbidity. ^d^Antibiotic prophylaxis may be considered for the first three cycles of therapy to reduce the risk of infection. ^e^Thromboprophylaxis should be considered for all patients receiving pomalidomide plus low-dose dexamethasone to reduce the risk of VTE.

The pomalidomide plus low-dose dexamethasone regimen represents an important new treatment option for patients with RRMM refractory to lenalidomide and bortezomib. The improvements in PFS and OS indicate that pomalidomide plus low-dose dexamethasone has the potential to change the course of the disease,^16^ and corresponding improvements in patient condition^16, 87^ are helping to expand therapeutic options after treatment with pomalidomide plus low-dose dexamethasone. These encouraging results highlight the importance of the appropriate use of pomalidomide, including adequate dose and prophylaxis for neutropenia, infection and VTE (Figure 6). Addressing these factors can help haematologists and oncologists maximise efficacy and minimise safety risks during pomalidomide treatment.

The optimal sequence and choice of agents in RRMM has not yet been established, and treatment decisions are left to the treating physician based on the needs of the individual patient and the availability of drugs. For patients who have exhausted lenalidomide- and bortezomib-based therapies, pomalidomide plus low-dose dexamethasone is an effective treatment option. Evidence suggests that pomalidomide is equally effective in patients whose last therapy was lenalidomide or bortezomib.^16^ Pomalidomide plus low-dose dexamethasone could be an option for second-line therapy in patients with disease that failed first-line therapies with a combination of proteasome inhibitors and immunomodulatory drugs such as lenalidomide, bortezomib and dexamethasone.

More data are needed on the use of pomalidomide plus low-dose dexamethasone in certain subpopulations of patients with RRMM, including those with renal impairment or unfavourable cytogenetic features. The ongoing MM-008 and MM-013 studies will provide much-needed data on the use of pomalidomide in patients with renal impairment (including dialysis patients) that will help develop an evidence-based dose-modification strategy for pomalidomide in this setting.^89, 93^ Ongoing studies, IFM 2010-02 (NCT01745640) and MM-010 (NCT01712789), will provide further data on the impact of cytogenetic features and may help identify markers of response. Currently, data on the role of cereblon as a biomarker are not strong enough to recommend its use in clinical practice.

Novel pomalidomide-based regimens continue to be explored. Preliminary evidence from phase I/II trials indicates that the combination of pomalidomide, cyclophosphamide and prednisone is feasible in RRMM^100^ as is the combination of clarithromycin, pomalidomide and dexamethasone^101^ and pegylated liposomal doxorubicin, pomalidomide and dexamethasone.^102^ Trials evaluating proteasome inhibitors in combination with pomalidomide and dexamethasone are also underway.^103, 104^ These studies will help further define the role of pomalidomide in the management of RRMM.

## Figures and Tables

**Table 1:** Pharmacokinetic properties of lenalidomide and pomalidomide^17, 88^

*Pharmacokinetic property*	*Lenalidomide*	*Pomalidomide*
Absorption (time to *C*_max_, h)	0.5–2	2–3
		
*Elimination (median plasma half-life, h)*		
Healthy volunteers	∼3	∼9.5
Myeloma patients	∼3–5	∼7.5
		
Excretion (% excreted unchanged in urine)	82	2
		
*Key drug–drug interactions*		
P-gp substrate	Yes; monitor closely if co-administered with P-gp inhibitors^a^	Yes; but no clinically relevant effect seen when co-administered with the P-gp inhibitor ketoconazole
CYP inhibitor/inducer	No; unlikely to affect exposure of other drugs	No; unlikely to affect exposure of other drugs
CYP substrate	No	Yes; monitor closely if co-administered with strong CYP1A2 inhibitors^b^

Abbreviations: CYP, cytochrome *P*450; P-gp, P-glycoprotein.

a Common P-gp inhibitors include cyclosporine, clarithromycin, itraconazole, ketoconazole, quinidine and verapamil.

b Common strong inhibitors of CYP1A2 include ciprofloxacin, enoxacin and fluvoxamine.

## Notes

MAD had a consultant role for, and has received honoraria from, Celgene. XL has received honoraria from Janssen-Cilag, Celgene Corporation, Amgen, Novartis, Onyx, LeoPharma and Sanofi-aventis. AP had a consultant role for, and has received honoraria from, Amgen, Bristol-Myers Squibb, Celgene, Janssen-Cilag, Millennium and Onyx. PM had a consultancy or advisory role and has received honoraria from Celgene, Millennium and Janssen. MD is an advisory board member of, and has received honoraria as a speaker from, Celgene. MC is an advisory board member of and a speakers' bureau member of Janssen and Celgene and has received honoraria from Millennium, Amgen, Bristol-Myers Squibb and Onyx. HL has received honoraria from, and is a speakers' bureau member of, Celgene, Mundipharma and Ortho Biotech. GJM received payment for lectures including service for speakers' bureaus from Novartis, Celgene and Ortho Biotech. FED has served as a speakers' bureau member of Celgene and Ortho Biotech and has participated in advisory boards of Celgene, Ortho Biotech and Novartis. PS is an advisory board member of Celgene, Janssen-Cilag and Onyx and has received research funding from Celgene and Janssen-Cilag. SAS is an advisory board member of, and has received honoraria as a speaker from, Celgene and NAPP Pharmaceuticals. SZ has received compensation as a scientific advisory board member of Celgene, Millennium and Janssen-Cilag. MH has nothing to declare. KW has received a research grant from Celgene and had a consultant role for, and has received honoraria from, Celgene and Janssen-Cilag. MVM is an advisory board member of Onyx Pharmaceuticals, GenMab, Janssen Pharmaceuticals and Millennium; she is also a member of the speaker's bureau for Celgene, Janssen Pharmaceuticals, Millennium, Mundipharma and Novartis. TF is an advisory board member of Janssen-Cilag, Celgene, Amgen, Novartis, Onyx and Millennium and is a speakers' bureau member of Janssen-Cilag and Celgene. JFSM has received compensation as a scientific advisory board member from Millennium, Celgene, Janssen-Cilag, Novartis, Onyx, BMS and MSD.
